# How access to long-term care affects short-term care

**DOI:** 10.1186/s12913-026-15163-0

**Published:** 2026-07-22

**Authors:** Ismail Aydemir, René Bekker, Dennis Moeke, Bianca M. Buurman, Rob D. van der Mei

**Affiliations:** 1 Department of Mathematics, Vrije Universiteit Amsterdam, Amsterdam, The Netherlands; 2 Stochastics Group, Centrum Wiskunde & Informatica, Science Park 123, Amsterdam, 1098XG The Netherlands; 3 Department of Medicine for Older People, Amsterdam Public Health Research Institute, Amsterdam UMC, Vrije Universiteit Amsterdam, Amsterdam, The Netherlands; 4 Research Group Logistics and Alliances, HAN University of Applied Sciences, Arnhem, The Netherlands; 5 Section of Geriatric Medicine, Department of Internal Medicine, Amsterdam Public Health Research Institute, Amsterdam UMC, University of Amsterdam, Amsterdam, The Netherlands

**Keywords:** Nursing homes, Long-term care, Short-term care, Discrete-event simulation, Capacity management

## Abstract

**Objective:**

Healthcare systems face growing pressure due to rising demand, population aging, and staffing shortages. In the Netherlands, limited access to long-term care often results in clients being admitted to short-term care while awaiting nursing home placement, increasing strain on these services. Over 50% of nursing home clients are admitted via short-term care, an outcome considered undesirable. This study takes a macro-level approach to assess to what extent more efficient nursing home bed allocation can reduce waiting times and ease pressure on the system.

**Methods:**

Modeling admission trajectories of older adults in need of nursing home care across long- and short-term care settings in the Netherlands, using discrete-event simulation. The model simulates client arrivals, admissions to and discharges from both short-term care and long-term care. We compare the current decentralized admission policy to a centralized admission policy, using average waiting times and fraction of short-term care admissions as performance metrics.

**Results:**

A centralized admission policy substantially reduces waiting times and short-term care admissions. For the base case scenario, with 90% regional bed occupancy, waiting times drop from 336 to 35 days, and short-term care admissions decrease from 45% to 4.5%. When demand variation across nursing homes is higher, these reductions can be even larger.

**Conclusions:**

Results show that long waiting times and uneven demand across nursing homes increase short-term care admissions and place additional pressure on the system. Efficient bed allocation may help reduce this pressure. Its effectiveness is largest when the system is working close to its full capacity, while it’s less affected when it’s running far below or well beyond that point. Additionally, an increase in *non long-term care* client admissions to the short-term care triggers a multiplier effect, further straining the system. These dynamics highlight the interdependency between long-term care and short-term care.

**Supplementary Information:**

The online version contains supplementary material available at https://doi.org/10.1186/s12913-026-15163-0.

## Introduction

The pressure on the healthcare system has increased significantly in recent years, both in the Netherlands and across the globe. While population aging is a significant driver of this trend, staffing shortages also play an important role [1, 2]. Among older adults, not only is the population aged 65 and older growing, but people are also living longer, resulting in a rising share of those aged 80 and above. This phenomenon, known as double aging, places additional strain on long-term care (LTC) services such as nursing homes, especially as capacity expansion remains limited and government policies focus on supporting people to age in place for as long as possible [3–6]. However, research shows that delaying nursing home placement – for instance, by living longer at home – increases the risk of hospitalization [7].

In the Netherlands, those waiting for nursing home placement often face long waiting lists. As a result, many clients are initially admitted to short-term care (STC) facilities, these clients no longer follow the regular care trajectory from home to nursing home. Instead, they are admitted through prioritization to the first available nursing home bed via STC pathway. Over 50% of clients awaiting a nursing home bed are eventually placed through STC, often because their health deteriorates or their informal caregiver can no longer provide adequate support [8]. Regional healthcare professionals confirm this trend, pointing out that the majority of their clients are eventually placed in non-preferred nursing homes through such emergency-driven pathways [9]. These observations suggest that limited access to LTC may contribute to increased pressure on STC services. While expanding LTC capacity may appear to be a logical solution from a capacity-management perspective, it is often restricted due to policy constraints and staff shortages [10].

Limited access is not solely due to insufficient capacity; inefficiencies in admission and placement procedures may also contribute to delays [11, 12]. A recent study on waiting-list management in nursing homes showed that waiting times can be reduced through more efficient organization of admissions and placements [13]. This highlights the importance of system design and process efficiency in achieving timely and effective care delivery. Following this line of research, we explore how limited access to LTC contributes to increased pressure on STC facilities. In particular, we assess to what extent reducing waiting times through more efficient allocation can reduce excess demand and relieve the pressure on the system. Whereas the reduction of waiting times in LTC using an allocation algorithm have also been addressed in [13], their approach did not account for the prioritized STC pathway. Therefore, in this study, we develop a discrete-event simulation to approximate the system at a macro-level. Using the Amsterdam region in the Netherlands as a case study, the model integrates LTC and STC into a single model framework and analyzes how they affect one another.

## Background

### Long-term care (LTC)

In the Netherlands, nursing homes generally provide care to two primary groups of clients. One group consists of individuals requiring geriatric rehabilitation or post-acute care following hospital discharge. The other group, which is the focus of this study, includes those in need of long-term institutional care, often for the remainder of their lives. Access to nursing home care in the Netherlands is regulated under the country’s long-term care system. Individuals must first be assessed and approved by *Centrum Indicatiestelling Zorg* (CIZ) [14], an independent government agency responsible for evaluating whether a person’s condition requires constant care or supervision, 24 h a day. Upon approval, CIZ assigns a care package that specifies the level of care intensity the client is eligible to receive. Clients can then seek placement in any nursing home that offers availability matching their assigned care package. This structured approach ensures that nursing home resources are allocated according to individual care needs within a standardized system [15].

### Short-term care (STC)

In this study, we define STC as any form of hospital or institutional care provided for a short period of time, generally not exceeding a few months. This is different from LTC, which usually provides care for many months to years or until the end of a person’s life. The types of STC included in this study are short-term residential care [16], geriatric rehabilitation [17], and hospital-based care, including the emergency department, acute admission units, and clinical wards.

### System description

**Fig. 1:**
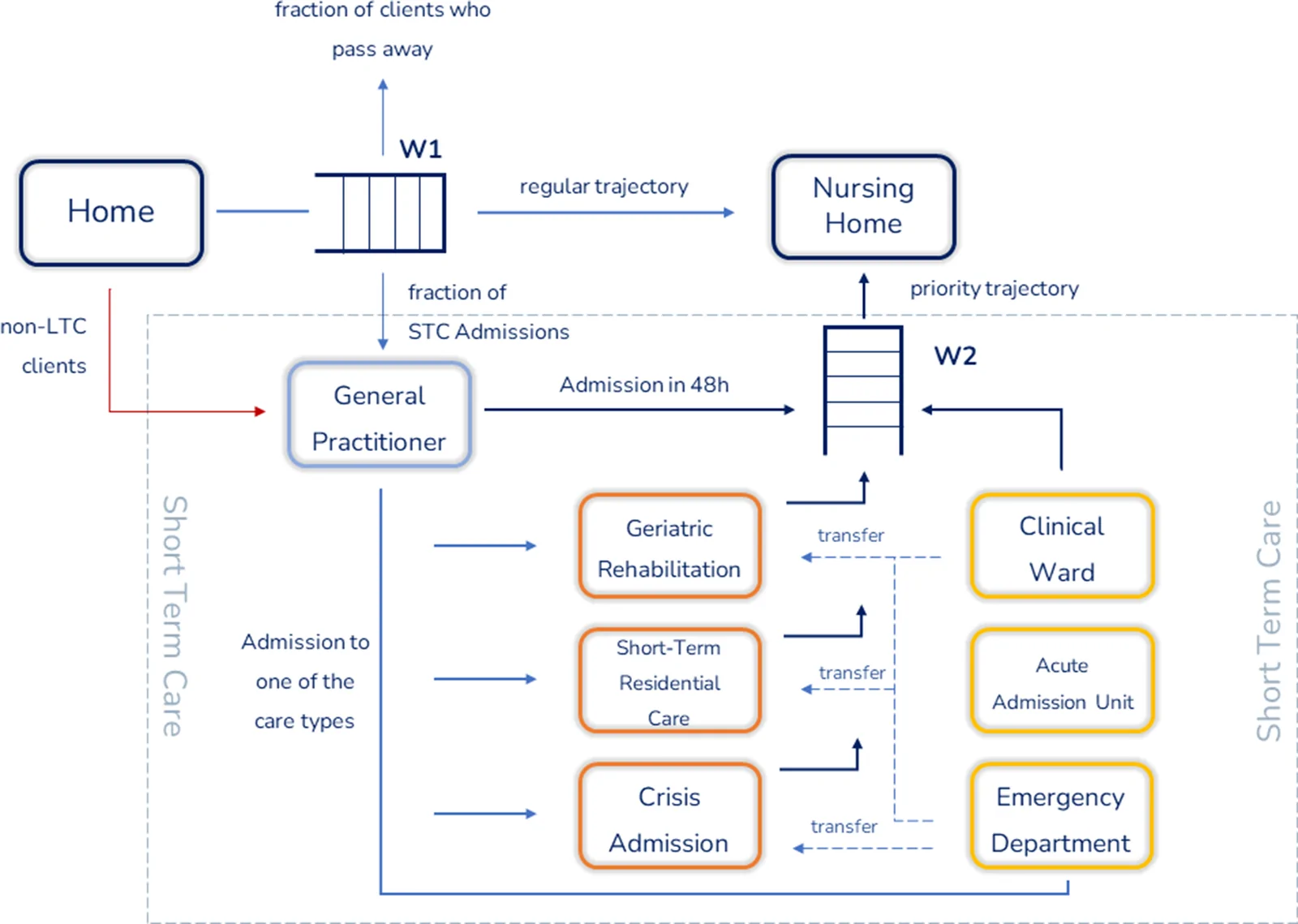
Schematic representation of pathways to nursing home admission. The regular trajectory represents clients with LTC approval waiting for an available bed, while the priority trajectory follows clients who enter through an STC facility. Additionally, non-LTC clients, who do not have a LTC approval, may also enter the system through short-term care and eventually be placed in a nursing home

Figure 1 illustrates the pathways to a nursing home. This macro-level perspective allows us to understand the dynamics and interactions within the overall system. Specifically, Fig. 1 illustrates both the regular and prioritized pathways toward nursing home admission. Clients who have been approved by CIZ to receive LTC in a nursing home remain at home while waiting for placement. This is represented by W1, the nursing home waiting list. During this waiting period, a client’s condition may deteriorate, or unforeseen events may occur, leading to admission into one of the STC services shown in Fig. 1. Alternatively, clients may pass away while awaiting nursing home admission. In addition to the previously mentioned STC types, the system also includes *crisis admissions.* These are designed for clients who require bed-based care within 24 h. Such clients are temporarily placed in crisis beds and are expected to be transferred to a nursing home within seven days. Furthermore, Fig. 1 includes a direct admission pathway for clients who must be placed in a nursing home within 48 h but do not require bed-based care in an STC setting. Once admitted to STC, clients may transition through various STC services or be admitted directly to a nursing home upon completion of their stay. Clients following this route are placed into a *virtual queue* (W2) and are effectively prioritized over those on the regular waiting list (W1). This is due to the fact that STC beds are not intended for prolonged use. Although W2 does not represent an official waiting list, it reflects the practical reality that these clients are awaiting nursing home placement while occupying a STC bed. As such, they are admitted to the first available nursing home bed ahead of those still waiting at home.

Additionally, the model does not capture the full sequence of intermediate events leading up to multiple STC admissions. Instead, it focuses solely on the final STC admission prior to nursing home placement. Hence, the return of clients from STC to home is not included in the system design. Figure 1 also includes a red line extending from the home icon, representing non-LTC arrivals. These clients have care needs similar to LTC clients but are not yet part of the system, which may be due to postponed LTC applications for financial reasons, ineligibility (e.g., medical conditions that are not yet severe enough), or other reasons. When an unforeseen event occurs (such as a deterioration in health), however, they may still enter the care chain by being admitted to an STC facility, after which they are directed to a nursing home through the priority pathway. Although this group is usually relatively small, it is important to include them in the system design, as they place additional pressure on available resources outside of the regular LTC client flow.

## Methods

### Current admission policy

In the Netherlands, clients are allowed to register only for a single preferred nursing home and wait at home until placement becomes available. In the absence of a centralized system for managing bed availability, regional office staff must contact each nursing home individually to check for availability. As a result, the allocation is based on a set of independent waiting lists, each specific to a single nursing home [13].

### Centralized admission policy

Under a centralized admission policy, a single shared waiting list is maintained across all nursing homes in a given region. Clients are eligible for all available beds and are placed according to a first-come, first-served principle. This approach can be understood as a form of resource pooling. Compared to a system with separate waiting lists for each nursing home, a centralized system is generally more efficient and leads to shorter waiting times [18]. As mentioned above, the approach in [13] involves developing an allocation algorithm as an alternative to the current decentralized policy. The authors report that the efficiency gains achieved with their method are close to a centralized policy. More broadly, while the centralized policy examined in this study does not account for client preferences, prior research has shown that granting clients even minimal choice can improve system performance similarly to a centralized policy [19, 20]. Accordingly, in our study we approximate micro-level dynamics through a centralized policy, which serves as a proxy at the macro level.

### Data

The data used in this study is derived from a recent study on the optimized allocation of clients to nursing homes in the Amsterdam region [13]. As shown in Table 1, the dataset includes information on the number of nursing homes, total bed capacity, average length of stay, and average patience duration (average time until a client passes away or is admitted to the STC). Due to the growing population of older adults, it is expected that demand for nursing homes will continue to increase in the years to come. To account for this, we used several values for the arrival rates, incorporating the rising demand into the model. The arrival rates in Supplementary Table 1 are estimated based on different occupancy levels. The occupancy level refers to the percentage of beds occupied across all nursing homes within the region. Additionally, discussions with regional healthcare professionals revealed that demand is not evenly distributed across nursing homes: some facilities are more popular and experience higher demand than others. For this reason, we also included arrival rates reflecting uneven demand among nursing homes. The arrival rate representing the base case scenario corresponds to the *two-times* uneven demand at a 90% occupancy level. Additional information about the uneven demand scenarios can be found in the Supplementary Material.

**Table 1:** Overview of input parameters for the simulation model for the Amsterdam region, the Netherlands

Parameter	Value
Number of Nursing Homes (for Amsterdam)	39
Number of Beds (per nursing home)	20
Average Length of Stay (in days)	666
Average Patience Duration (time till final STC admission, in days)	666

Given that each nursing home has 20 beds, the total number of beds in the region is 780. The average length of stay for a nursing home client is 666 days, which is also the average time they are willing to wait for a bed. This patience duration is used in the model to capture queue abandonments, after which clients may transition to an STC facility

### Simulation

Discrete-event simulation (DES) is a computational modeling technique that represents a system as a sequence of time-based events, where each event represents a change within the system [21, 22]. In this study, such events represent client arrivals, admissions to STC, discharges from STC and nursing homes. The development of a DES model requires a thorough understanding of the system’s structure and dynamics. Hence, Fig. 1 forms the basis for the development of the model. Once the model is built, the data presented in Table 1 and Supplementary Table 1 serve as input parameters. We then use the model to simulate the system’s performance over time and compare the current (decentralized) admission policy with a centralized admission policy, using average waiting times and STC admissions as evaluation metrics. The resulting output can be analyzed to gain insights into client flows and capacity utilization. Furthermore, we model arrivals as a Poisson process, and both patience and service durations are exponentially distributed, as this is common in healthcare system modeling. In large populations, arrivals have often been shown to approximate a Poisson process. For a detailed justification of this assumption, see [23, 24]. Furthermore, since the model represents a system with a large number of beds, selecting a non-exponential distribution for the length of stay would have a negligible impact on the results [25]. Such effects are generally observed in systems with a small bed capacity.

The simulation was built from scratch using MATLAB [26], without the use of any pre-built simulation tool. Hence, the same model can be implemented using any other software. The model simulates 100 batches, with each batch consisting of 20,000 runs, where the first 6,000 runs serve as the warm-up period. We have designed, implemented, and executed the model in line with the STRESS-DES guidelines [27]. For further details on the model, see the Supplementary Material.

## Results

### Current admission policy

**Fig. 2:**
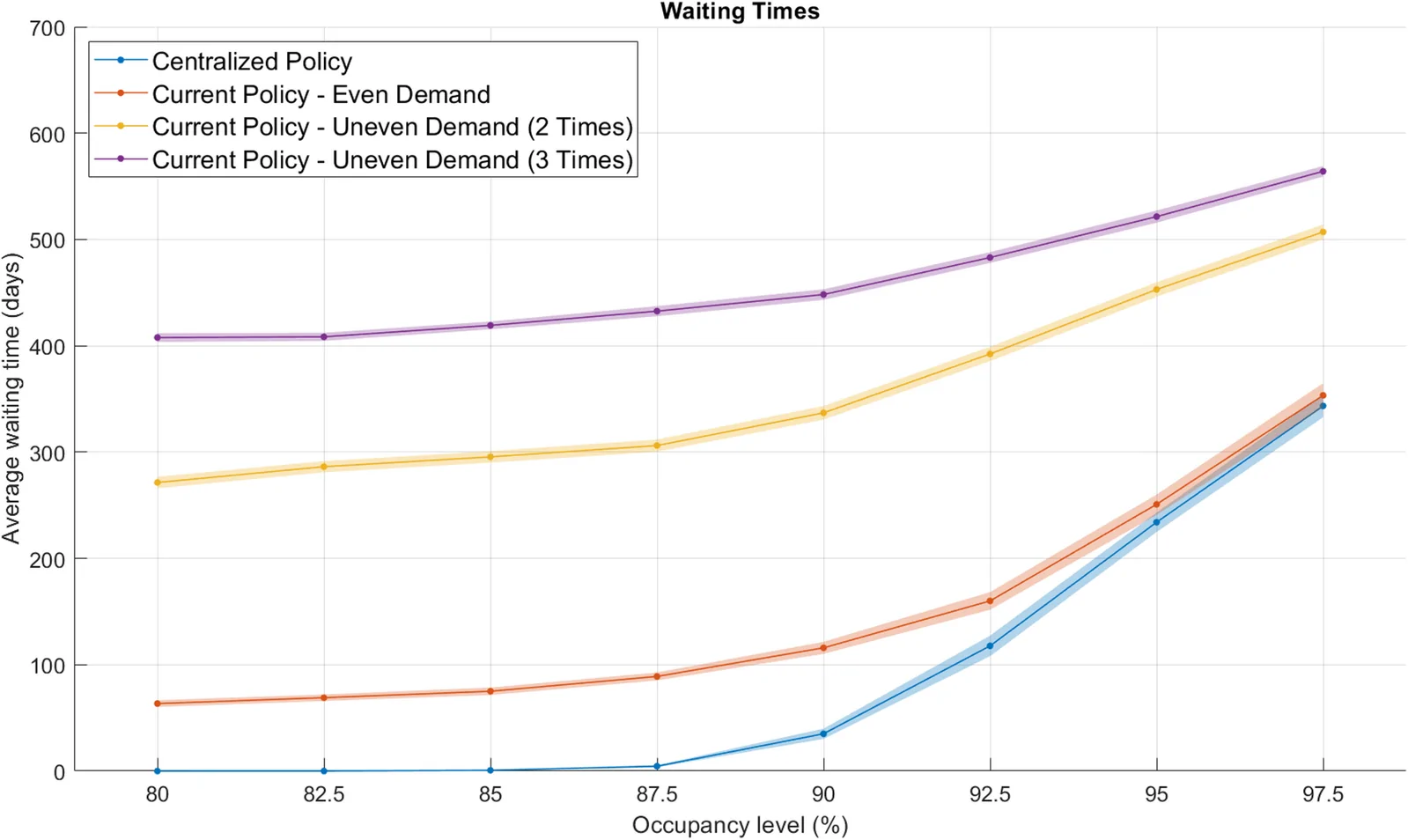
This figure illustrates the average waiting times for nursing homes under the current decentralized policy across different demand levels, as well as the corresponding waiting times under a centralized policy, shown by the blue line. The base case is defined as the decentralized policy with two-times uneven demand at 90% occupancy, represented by the yellow line. All other lines represent alternative scenarios

Figure 2 illustrates the average waiting times under the current decentralized policy. It includes the scenario with evenly distributed demand across nursing homes, which is unlikely in practice, and is used as a benchmark scenario for comparison. It also includes scenarios with uneven demand, where arrival rates vary by a factor of two and three across nursing homes, compared to the evenly distributed scenario.

In the case of evenly distributed demand, waiting times begin to increase more rapidly when regional bed occupancy exceeds 90%. In contrast, under uneven demand across nursing homes, waiting times are already significantly higher at lower occupancy levels. In the base case scenario, waiting times can reach nearly 300 days at a regional occupancy level of 80%. In the three-times uneven demand scenario, waiting times increase to as much as 400 days at the same occupancy level. This is significantly higher than in the scenario where demand is evenly distributed, even when occupancy levels are close to 100%.

**Fig. 3:**
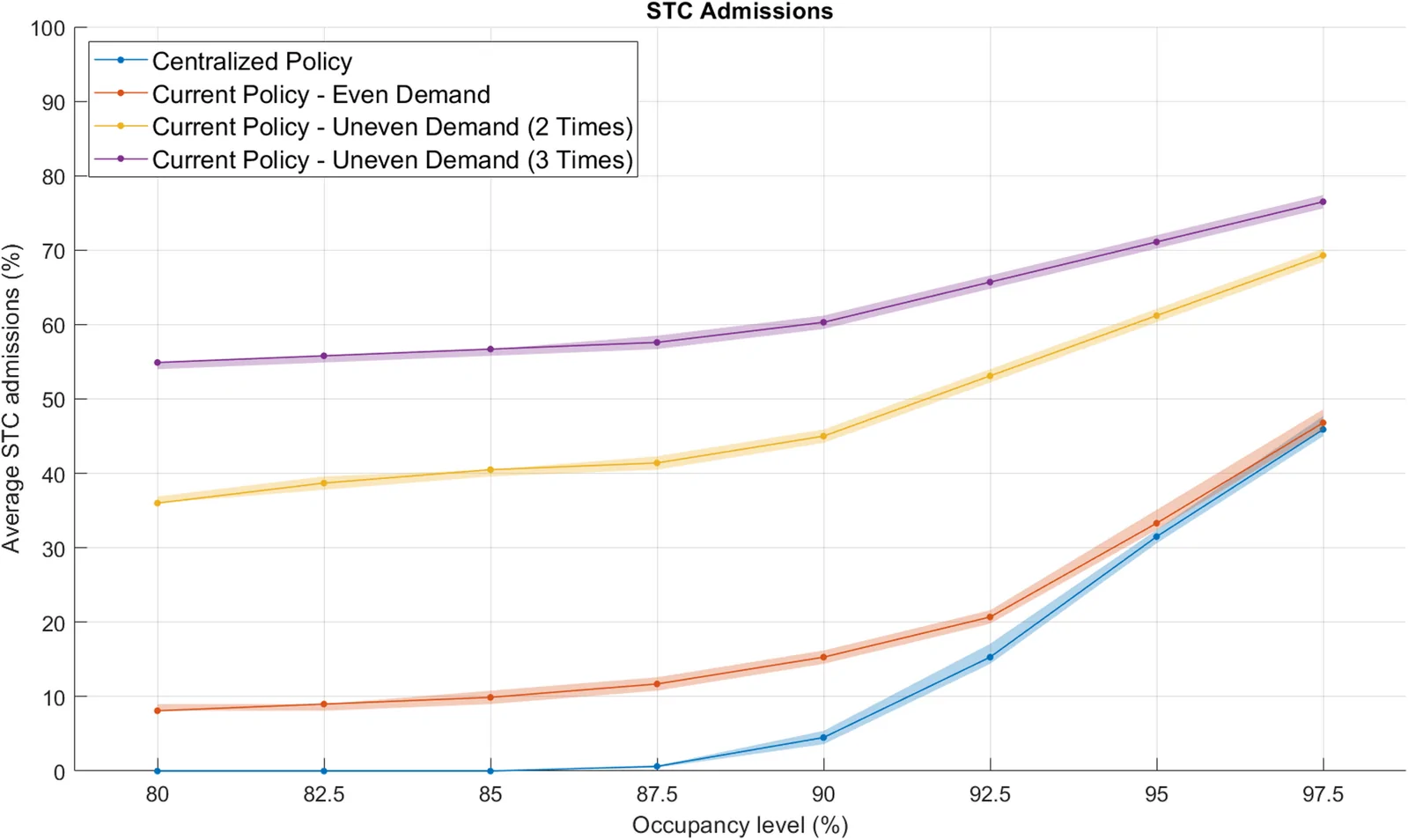
This figure illustrates the average percentage of STC admissions among LTC clients who, after waiting too long for a nursing home bed, leave the queue and are admitted to STC, and eventually being placed in a nursing home via the priority trajectory. Results are shown for the current decentralized policy across different demand levels, as well as for the corresponding outcomes under a centralized policy, shown by the blue line. The base case is defined as the decentralized policy with two-times uneven demand at 90% occupancy, represented by the yellow line. All other lines represent alternative scenarios

Figure 3 shows the percentage of STC admissions consisting of clients from the regular waiting list (W1 in Fig. 1) who are awaiting nursing home placement and are eventually admitted via STC. The same underlying dynamics identified in Fig. 2 are also present here. Under the evenly distributed demand scenario, the percentage of clients admitted to nursing homes via STC reaches 50% when the regional bed occupancy level becomes 97.5%. In contrast, under the two-times uneven demand scenario, the number of STC admissions is 1.5 times higher at the same occupancy level. A similar pattern of increase is also observed in waiting times. Additional scenarios involving higher variation in demand between nursing homes are reported in Supplementary Tables 2 and 3. In these cases, average waiting times can reach up to 600 days, and nursing homes placements via STC up to 80%.

### Centralized admission policy

The blue line in Figs. 2 and 3 represents the scenario under a centralized admission policy. Under this policy, both average waiting times and STC admissions remain close to zero as long as the occupancy level does not exceed 90%. At an occupancy level of 90%, the average waiting time is 35 days, and the fraction of STC admissions is 4.5%. However, when a large portion of beds within the region is occupied, such as at an occupancy level of 97.5%, waiting times exceed 300 days and STC admissions rise to 45%.

### Comparison

When comparing the current policy scenarios with the centralized admission policy, substantial reductions are observed in both waiting times and STC admissions. Notably, the reduction factor is similar for both metrics across scenarios. For the base case scenario, implementing the centralized admission policy reduces the average waiting time from 336 days to 35 days and STC admissions from 45% to 4.5%, both indicating substantial improvement in system performance. For occupancy levels below 90%, the reduction tends to be even higher. However, as bed occupancy approaches 100%, the impact of centralized admissions diminishes. At an occupancy level of 97.5%, the average waiting time under the two-times uneven demand scenario is approximately 500 days, and STC admissions are around 69%. With a centralized policy, these are reduced to approximately 340 days and 46%. While this represents a meaningful improvement in absolute terms, both the waiting time and STC admissions remain high from a practical standpoint.

### Non-LTC clients

**Fig. 4:**
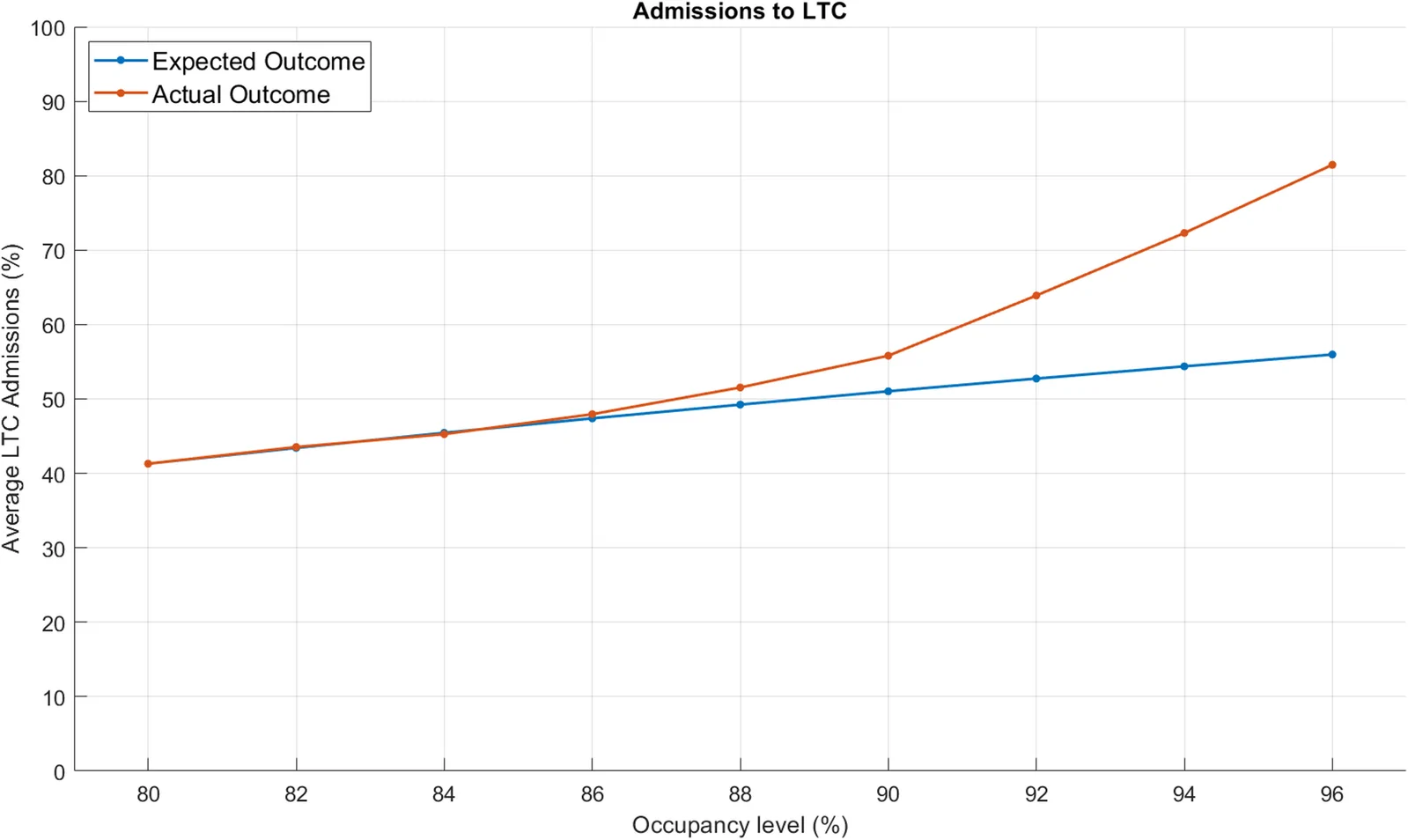
This figure shows the effect of an increasing number of non-LTC clients, while the regular flow of LTC clients remains unchanged, on admissions to nursing homes. The blue line represents the expected outcome, whereas the red line shows what actually occurs

Figure 4 illustrates the effect of increasing the arrival rate of non-LTC clients (represented by the red line in Fig. 1) on nursing home admissions, while keeping the arrival rate of LTC clients constant. A practical scenario in which this may occur is when the number of clients rejected by CIZ for LTC increases, or when a growing number of clients postpone their LTC applications. The blue line in Fig. 4 reflects the expected outcome, where the LTC arrival rate is set at a fixed value corresponding to the *two-times* uneven demand at an occupancy level of 80%. In this case, an increase in non-LTC arrivals, while STC admissions of regular LTC clients remain unchanged, would suggest that total LTC admissions should increase proportionally with the increase in non-LTC arrivals. However, the red line shows a different pattern. Once the occupancy level exceeds 90%, the number of LTC admissions via STC increases by more than the additional inflow of non-LTC clients. Additionally, Supplementary Fig. 1 shows STC admissions from LTC clients under increased non-LTC arrivals, both under the current policy and centralized admission policy. In Supplementary Table 4, the arrival rates used to model the effect of non-LTC clients can be found.

## Discussion

The objective of this study was to explore how access to LTC affects STC, where access is expressed in terms of waiting times. More specifically, we examined the extent to which long waiting times for nursing homes put pressure on the entire system. To capture the macro-level dynamics of the system, we used a DES model. The findings of this study quantify how prolonged waiting times for nursing homes lead to increased STC admissions, resulting in emergency-driven nursing home placements. However, this rise in STC admissions reflects excess demand in the system, demand that is not inevitable and could potentially be prevented. Namely, our results indicate that efficient bed allocation not only reduces waiting times for nursing homes but also relieves pressure on STC by decreasing emergency-driven admissions.

In short, improving access to LTC has system wide benefits. However, the extent of these benefits is dependent on bed occupancy levels within the region. When occupancy exceeds a certain level, for example 97.5%, the impact of optimal allocation becomes considerably smaller, as the underlying issue shifts from inefficiency to a lack of available capacity. In such cases, even if inefficiencies are present, the capacity shortage dominates the system and leaves little room for improvements in allocation. A centralized allocation is most effective in situations where capacity might be tight, but the number of beds is sufficient to handle the offered workload without the need to defer patients. When occupancy is near its maximum, capacity scarcity rather than allocation inefficiency becomes the primary constraint.

Furthermore, our results indicate that it is not only long waiting times themselves that put pressure on the system, but also uneven demand across nursing homes, where certain homes are more popular than others. This imbalance of demand between nursing homes causes waiting times at some locations to reach extreme levels. Consequently, STC admissions rise beyond what would be expected under a more evenly distributed demand.

In addition, this study underscores that even when access to LTC appears adequate, additional pressure from STC, such as an increase in non-LTC arrivals, can trigger a snowball effect. This effect occurs because non-LTC clients entering STC follow a priority trajectory and effectively bypass the regular queue for nursing homes. As a consequence, clients on the regular trajectory experience longer waiting times, which in turn leads to an increase in STC admissions among LTC clients. This demonstrates that an increase in non-LTC arrivals triggers a multiplier effect on STC admissions, contrary to what would be expected if LTC and non-LTC clients were independent of each other.

Although prior research has explored the effects of admission policies on access to healthcare services [11, 12], particularly in the context of optimizing nursing home bed allocation [10], most studies do not model the entire system as a set of interacting elements. This highlights a current research gap and underscores the relevance of this study, as LTC and STC are not independently functioning elements but are, in fact, dependent and complementary to each other. One study examined how LTC eligibility influences the likelihood of hospital admission [8]. However, it focused only on a single care type and did not consider the broader STC as a whole. In this regard, our findings support previous research while contributing new insights through a system level analysis of the interactions between LTC and STC. By modeling these interactions, the study reveals how pressure in one part of the system affects the other.

### Model limitations

A limitation of this study is the unavailability of data on arrivals from the LTC waiting list into STC. An additional limitation of our approach is that, by using a macro-level approximation, the model does not account for individual client preferences regarding nursing home placement, such as location, quality, or cultural fit. However, this simplification is justifiable, as a similar approach is taken in [13], where such preference-related elements are incorporated in a micro-level model. Our study builds on their work by approximating the detailed preference factors at a macro-level. Moreover, the primary objective of our study is not solely to quantify the potential gains of a centralized admission policy (or any other optimal policy), but also to examine how reducing waiting times influences the inflow to STC, also known as the priority trajectory. Furthermore, since nursing homes differ in practice, for example in the quality of care they provide, resulting in higher or lower levels of demand, there is heterogeneity across facilities. Since our approach is at the macro-level and does not explicitly incorporate micro-level heterogeneity, we address this variation by evaluating multiple scenarios with uneven demand levels.

In short, the centralized policy in our model abstracts from the detailed mechanisms by which patients are allocated to nursing homes, resulting in an idealized scenario. As such, implementing such a policy may involve several practical challenges. Although the policy addresses inefficiencies at the system level, it may not always be beneficial from the perspective of individual nursing home organizations, particularly from a financial standpoint. Consequently, persuading all organizations within a region to adopt a centralized policy may prove challenging. Nevertheless, even partial collaboration among a limited number of organizations may still yield positive system-level benefits.

Furthermore, geographic factors may reduce the practical feasibility of a centralized policy. This is particularly relevant in rural areas, where nursing homes are fewer in number and more geographically dispersed. Even if care providers agree to operate under such a policy, it may be less desirable from the clients’ perspective. In urban areas, admission to a nursing home located relatively far from home may still be acceptable, as distances between facilities are generally smaller. In rural areas, however, clients may have to be admitted to a nursing home substantially farther away than would typically be the case in an urban setting. This may be undesirable for clients and could therefore limit the practical implementation of a centralized policy.

However, since the detailed micro-level model presented in [13] demonstrates near-optimal reductions in waiting times, using a centralized admission policy as a proxy at the macro level is a reasonable assumption. While practical outcomes may vary slightly, the model captures the fundamental patient flow dynamics at the system level. In particular, one of the main contributions of this study is showing that a centralized admission policy can reduce STC admissions. This insight is especially relevant given the macro-level nature of the model, which does not model waiting times at individual nursing homes in detail. This also creates opportunities for future research, where a hybrid approach could be used to incorporate all relevant details of both LTC and STC, resulting in a more realistic and comprehensive representation of the system.

## Conclusions

This study explored access to LTC and its impact on STC through the use of a discrete-event simulation. We evaluated whether optimizing the current admission policy could improve access to nursing homes and, in turn, relieve pressure on the system. The simulation results show that long waiting times, and thus limited access, in LTC increase pressure on STC. Efficient bed allocation in LTC can serve as a useful instrument to relieve this pressure. Specifically, in the base case scenario waiting times can be reduced by up to 300 days. In terms of system pressure, this translates to a 40% point reduction in STC admissions from LTC clients awaiting nursing home placement. However, it is also important to prevent system overload through proactive planning. At high bed occupancy levels, the system becomes primarily constrained by capacity shortages, and improving admission policies alone is not enough to ease the pressure. Policymakers should proactively assess current capacity levels and plan capacity scaling accordingly to avoid scenarios of excessive occupancy. Our results indicate that when some nursing homes are significantly more popular than others, this can lead to inadequate access at certain locations. In turn, this increases emergency-driven STC admissions, further straining the system. Therefore, demand across nursing homes should be balanced as evenly as possible. Additionally, the results show that an increase in the number of non-LTC clients leads to a snowball effect, placing additional pressure on the system, even when overall access to LTC appears adequate. Hence, it is essential to track the number of non-LTC client admissions to STC facilities, particularly those who are eventually placed in nursing homes with priority.

## Supplementary Information

Below is the link to the electronic supplementary material.

Supplementary Material 1

## Data Availability

All data generated or analysed during this study are included in this published article and its supplementary information files.

## Abbreviations

LTC: Long-term care
STC: Short-term care
CIZ: Centrum Indicatiestelling Zorg
DES: Discrete-event simulation
non-LTC: Non long-term care
