# Supplementary material for: How access to long-term care affects short-term care
Source: BMC Health Serv Res. 2026 Jul 22;26:1427. doi: 10.1186/s12913-026-15163-0 (PMC13682145; doi:10.1186/s12913-026-15163-0)
Supplement: Supplementary file 1 [file 12913_2026_15163_MOESM1_ESM.docx]

Supplementary Material

Arrivals

To account for rising demand and uneven distribution of arrivals across nursing homes, we estimated multiple arrival rate values based on regional bed occupancy levels (see Supplementary Table 1).

| Occupancy Levels 🡪 | 80% | 82.5% | 85% | 87.5% | 90% | 92.5% | 95% | 97.5% |
| --- | --- | --- | --- | --- | --- | --- | --- | --- |
|  | *Arrival Rates (per day)* | | | | | | | |
| Total Arrivals  (for Amsterdam) | 0.989 | 1.020 | 1.051 | 1.082 | 1.113 | 1.144 | 1.174 | 1.205 |
| Even Demand  (zero popularity) | 0.025 | 0.026 | 0.027 | 0.028 | 0.029 | 0.029 | 0.030 | 0.031 |
| 2 Times Uneven Demand  (base case) | 0.051 | 0.052 | 0.054 | 0.055 | 0.057 | 0.059 | 0.060 | 0.062 |
| 3 Times Uneven Demand | 0.076 | 0.078 | 0.081 | 0.083 | 0.086 | 0.088 | 0.090 | 0.093 |
| 6 Times Uneven Demand | 0.152 | 0.157 | 0.162 | 0.166 | 0.171 | 0.176 | 0.181 | 0.185 |

*Supplementary Table 1: Arrival rates for the simulation to account for rising demand and uneven distribution across nursing homes, based on regional bed occupancy levels.*

Demand

Based on discussions with regional healthcare professionals, it was noted that some nursing homes are significantly more popular than others, making an evenly distributed demand scenario across the region unlikely. Nevertheless, we included this scenario for comparison with the base case. The base case scenario represents a *tagged* nursing home where the arrival rate is two times that of the evenly distributed demand. This can be easily observed from the values in Supplementary Table 1. Although higher levels of demand variation are considered unlikely, they were included to explore potential outcomes under more extreme conditions. The arrival rates in these scenarios again represent a *tagged* nursing home receiving x-times higher demand compared to the evenly distributed case. See Supplementary Table 2 for average waiting times and Supplementary Table 3 for STC admissions across all scenarios.

| Occupancy Levels 🡪 | 80% | 82.5% | 85% | 87.5% | 90% | 92.5% | 95% | 97.5% |
| --- | --- | --- | --- | --- | --- | --- | --- | --- |
| *Policy* | *Average Waiting Time (days)* | | | | | | | |
| Centralized Admission | 0  (0 – 0) | 0.05  (0.02 – 0.09) | 0.72  (0.54 – 0.89) | 4.53  (3.55 – 5.51) | 35.06  (30.23 – 39.99) | 117.71  (108.05 – 127.36) | 234.03  (224.88 – 243.18) | 343.42  (332.86 – 353.97) |
| Current Admission – Even Demand | 63.48  (60.29 – 66.66) | 68.98  (65.91 – 72.06) | 75.04  (71.53 – 78.56) | 88.98  (85.24 – 92.74) | 115.88  (110.19 – 121.56) | 160.02  (151.70 – 168.32) | 250.86  (241.65 – 260.08) | 353.40  (341.97 – 364.83) |
| Current Admission –  2 Times (base case)  Uneven Demand | 271.40  (265.97 – 276.82) | 286.28  (281.04 – 291.54) | 295.55  (290.13 – 300.96) | 306.15  (300.42 – 311.87) | 336.96  (330.57 – 343.35) | 392.33  (385.59 – 399.07) | 452.97  (446.09 – 459.85) | 507.13  (500.18 – 514.08) |
| Current Admission –  3 Times  Uneven Demand | 407.76  (403.63 – 411.90) | 408.43  (404.60 – 412.27) | 419.21  (415.54 – 422.88) | 432.57  (427.81 – 437.33) | 448.20  (443.25 – 453.16) | 482.03  (477.95 – 488.11) | 521.51  (515.75 – 527.28) | 564.11  (559.16 – 569.07) |
| Current Admission –  6 Times  Uneven Demand | 526.47  (523.22 – 529.71) | 532.33  (529.55 – 535.11) | 532.09  (529.17 – 535.010 | 540.27  (537.37 – 543.17) | 547.37  (544.28 – 550.47) | 564.89  (562.64 – 569.14) | 588.51  (585.09 – 591.93) | 601.99  (598.85 – 605.14) |

*Supplementary Table 2: Simulation results of average waiting times, with 95% confidence interval, across scenarios with varying levels of demand distribution.*

| Occupancy Levels 🡪 | 80% | 82.5% | 85% | 87.5% | 90% | 92.5% | 95% | 97.5% |
| --- | --- | --- | --- | --- | --- | --- | --- | --- |
| *Policy* | *STC Admissions (%)* | | | | | | | |
| Centralized Admission | 0 (0 – 0) | 0  (0 -0) | 0  (0 – 0) | 0.63  (0.47 – 0.74) | 4.5 (4.1 – 5.4) | 15.3  (14.6 – 17.1) | 31.5  (30.4 – 32.9) | 45.9  (45 – 47.7) |
| Current Admission –  Even Demand | 8.1  (8.0 – 9.0) | 9  (8.8 – 9.7) | 9.9  (9.7 – 10.7) | 11.7  (11.5 – 12.5) | 15.3  (14.9 – 16.4) | 20.7  (19.9 – 22.1) | 33.9  (32.7 – 35.2) | 46.8  (45.9 – 48.6) |
| Current Admission –  2 Times (base case)  Uneven Demand | 36.7  (36.1 – 37.4) | 38.7  (38.2 – 39.3) | 40.5  (39.6 – 40.9) | 41.4  (41.0 – 42.2) | 45  (44.5 – 45.9) | 53.1  (52.3 – 53.8) | 61.2  (60.5 – 62.1) | 69.3  (68.5 – 70.0) |
| Current Admission –  3 Times  Uneven Demand | 54.9  (54.45 – 55.26) | 55.8  (55.2 – 55.9) | 56.7  (56.3 – 56.9) | 57.6  (57.4 – 58.4) | 60.3  (60.2 – 61.1) | 65.7  (65.3 – 66.5) | 71.1  (70.4 – 71.6) | 76.5  (76.2 – 77.2) |
| Current Admission –  6 Times  Uneven Demand | 72.1  (71.9 – 72.3) | 72.7  (72.5 – 72.9) | 73  (72.9 – 73.2) | 73.9  (73.7 – 74.1) | 75.06  (74.8 – 75.3) | 78.3  (77.6 – 78.2) | 80.6  (80.4 – 80.9) | 83.3  (83.1 - 83.6) |

*Supplementary Table 3: Simulation results of STC admissions, with 95% confidence interval, across scenarios with varying levels of demand distribution.*

Impact of non-LTC clients

Supplementary Figure 1 shows STC admissions from LTC clients under increased non-LTC arrivals, comparing both the current policy and the centralized admission policy. Notably, admissions begin to rise once the occupancy level exceeds 90%. This turning point is also visible in Figure 4, reflecting the onset of the multiplier effect. At this point, the increase in non-LTC admissions begins to affect the regular LTC admission trajectory, displacing those clients into STC. This, in turn, leads to higher LTC admissions via STC, further straining the regular pathway and creating a snowball effect.

*Supplementary Figure 1: This figure shows the effect of an increasing number of non-LTC clients on STC admissions from the regular queue (W1 in Figure 1), while the regular flow of LTC clients remains unchanged. The red line represents the base case setting: the decentralized policy with two-times uneven demand. The blue line shows the corresponding outcome under the alternative centralized policy.*

Arrivals of non-LTC clients

| Occupancy Levels 🡪 | 80% | 82% | 84% | 86% | 88% | 90% | 92% | 94% | 96% |
| --- | --- | --- | --- | --- | --- | --- | --- | --- | --- |
|  | *Arrival Rates (per day)* | | | | | | | | |
| LTC clients | 0.051 | 0.051 | 0.051 | 0.051 | 0.051 | 0.051 | 0.051 | 0.051 | 0.051 |
| non – LTC clients* | 0.071 | 0.094 | 0.1175 | 0.141 | 0.1645 | 0.188 | 0.2115 | 0.031 | 0.235 |

*Supplementary Table 4: Arrival rates to model the effect of non-LTC clients. The LTC arrival rate represents the demand for nursing homes (corresponding to the arrival rate under the two-times uneven demand scenario with 80% occupancy, as shown in Supplementary Table 1). *The non-LTC arrival rate reflects the total demand across the region.*

Detailed Model Description

In this section, we describe the queueing models used to simulate both admission policies. Whereas Figure 1 only illustrates the client trajectories, a more detailed model visualization for the queueing models is provided in Supplementary Figure 2 and 3.

In Supplementary Figure 2, Q_1_ corresponds to queue W1 in Figure 1, representing the regular trajectory for clients with LTC approval, and Q_2_ corresponds to W2, representing the priority trajectory. Clients arrive to queue Q_1_ at rate λ_1_ (see arrival rates in Supplementary Table 1). They may abandon the queue if they are not served before their patience time γ, with parameter value given in Table 1. Once in service, clients have an average service duration of β (see Table 1), after which they leave the system.

Clients who abandon the queue either exit the system (for example, representing a decease) with probability *p* (where *p* = 0.1) or enter the *orbit* with probability *1−p*. The orbit represents the STC, where the length of stay (33 days in our model) is relatively short in comparison to the average nursing home stay. After leaving the *orbit*, clients either return to the initial nursing home for which they were waiting or are admitted to a bed in one of the remaining nursing homes in the region.

Q_1_

λ_1_

β

*Remaining*

*Nursing Homes*

Q_2_

Q_3_

λ_3_

β

*Tagged*

*Nursing Home*

Q_2_ - priority

γ

decease

*p*

*1- p*

orbit

γ

decease

*p*

*1- p*

orbit

λ_2_

*Supplementary Figure 2:* *Queueing network to model the decentralized admission policy.*

Arrivals of non-LTC clients (represented by the red line in Figure 1) are modeled with rate λ_2_, which is obtained using Equation 1. These clients enter queue Q_2_ and are placed as soon as a bed becomes available in the system. Note that queue Q_2_ has priority over clients in queues Q_1_ and Q_3_. Here, Q_3_ represents the remaining clients in the region waiting for admission to other nursing homes.

$$\mathrm{Occupancy}=\frac{{((\lambda}_{1}+\lambda_{3})\left( 1-p \right)+\lambda_{2})\beta}{c}$$

*Equation 1: Approximating the regional occupancy for the decentralized system. As a function of arrival rates, service duration, and system capacity.*

The arrival rate λ_3_ can be derived directly from the values in Table 1 (Total Arrivals). Given the total arrival, subtracting the known arrival rate λ_1_ yields the remaining arrival rate for Q_3_. All other parameter values in the lower part of the queueing network are identical to those defined earlier. The value obtained from Equation 1 must be smaller than 1, such that the network is stable. The total number of beds in the system, denoted by *c*, can be obtained from Table 1. Given the other parameter values, the value of λ_2_ can then be derived using Equation 1.

The centralized admission policy is simulated using the queueing network shown in Supplementary Figure 3. In this model, Q_1_ represents a single central queue for the entire region. The total arrival rate λ_1_, shown in Supplementary Table 1, corresponds to the combined arrival rates (λ_1_ + λ_3_) from Supplementary Figure 2. Q_2_ again represents the priority trajectory, and all remaining parameters are identical to those described for the decentralized model. Equation 2 can be used to derive the value of λ_2_ in this setting. As in the previous case, the stability condition requires that the result of Equation 2 is smaller than 1.

Q_2_ - priority

λ_1_

β

Q_1_

λ_2_

*Pool of All*

*Nursing Homes*

γ

decease

*p*

*1- p*

orbit

*Supplementary Figure 3: Queueing network to model the centralized admission policy.*

$$\mathrm{Occupancy}=\frac{{(\lambda}_{1}\left( 1-p \right)+\lambda_{2})\beta}{c}$$

*Equation 2: Approximating the regional occupancy for the centralized system. As a function of arrival rates, service duration, and system capacity.*
